# PBMCs gene expression predicts liver fibrosis regression after successful HCV therapy in HIV/HCV-coinfected patients

**DOI:** 10.3389/fphar.2024.1436198

**Published:** 2025-01-22

**Authors:** Ana Virseda-Berdices, Óscar Brochado-Kith, Juan Berenguer, Juan González-García, Leire Pérez-Latorre, Carmen Busca, Cristina Díez, Rafael Micán, Amanda Fernández-Rodríguez, María Ángeles Jiménez-Sousa, Salvador Resino

**Affiliations:** ^1^ Unidad de Infección Viral e Inmunidad, Centro Nacional de Microbiología, Instituto de Salud Carlos III, Madrid, Spain; ^2^ Centro de Investigación Biomédica en Red de Enfermedades Infecciosas (CIBERINFEC), Instituto de Salud Carlos III, Madrid, Spain; ^3^ Unidad de Enfermedades Infecciosas/VIH, Hospital General Universitario “Gregorio Maranón”, Madrid, Spain; ^4^ Instituto de Investigación Sanitaria del Gregorio Maranón, Madrid, Spain; ^5^ Unidad de VIH; Servicio de Medicina Interna, Hospital Universitario “La Paz”, Madrid, Spain; ^6^ Instituto de Investigación Sanitaria La Paz (IdiPAZ), Madrid, Spain

**Keywords:** HIV/HCV coinfection, gene expression, RNA-seq, PBMCs, liver stiffness, HCV treatment, sustained virological response

## Abstract

**Background:**

HCV eradication with antiviral treatment reduces hepatic disease, but some patients remain at risk of progression to cirrhosis despite HCV clearance. We aimed to examine the association between peripheral blood mononuclear cells (PBMCs) gene expression before HCV therapy and a pronounced decrease in the liver stiffness measurement (LSM) value in HIV/HCV-coinfected patients after HCV treatment and achievement of sustained virological response (SVR).

**Methods:**

We performed a retrospective study in 48 HIV/HCV-coinfected patients who started anti-HCV treatment with at least advanced fibrosis (LSM ≥9.5). Total RNA was extracted from PBMCs at baseline, and poly(A) RNA sequencing was performed. The outcome was an LSM reduction greater than 50% (LSMred>50%) about 48 weeks after HCV treatment.

**Results:**

Seven patients (14.5%) reduced LSM by over 50%. We found 47 significant differentially expressed (SDE) genes associated with reaching an LSMred>50% after achieving HCV eradication, 42 upregulated and 5 downregulated in the LSMred>50% group. Ten and five of these upregulated genes were classified into two significantly enriched KEGG pathways: cell cycle and progesterone-mediated oocyte maturation (q-value <0.05), respectively. Two SDE genes achieved excellent discrimination ability: NCAPG had an AUROC of 0.908, NHLRC1 of 0.879, and a logistic regression model with these two genes of 0.955.

**Conclusion:**

A pre-treatment gene expression signature in PBMCs was associated with liver fibrosis regression (LSMred>50%) after achieving HCV clearing with HCV therapy in HIV/HCV-coinfected patients, where two SDE genes (*NCAPG* and *NHLRC1*) showed the greatest predictive capacity, which could be used as a noninvasive marker of liver fibrosis regression.

## Graphical Abstract

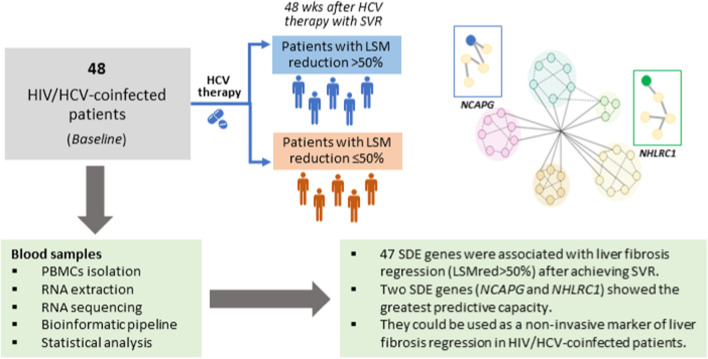

## 1 Introduction

Approximately 20%–30% of people with chronic hepatitis C progress to liver cirrhosis within approximately 25 years (Lingala and Ghany, 2015). Cirrhotic patients are at increased risk of developing End-stage liver disease and hepatocarcinoma (HCC) (Lingala and Ghany, 2015). Human immunodeficiency virus (HIV)/hepatitis C virus (HCV) coinfection accelerates liver disease progression, increasing liver-related events and co-morbidities (Ingiliz and Rockstroh, 2015).

The liver can regenerate in response to injury by increasing the rate of hepatocyte mitosis and differentiating stem cells, progenitor cells, and extrahepatic stem cells into hepatocytes and cholangiocytes (Hora and Wuestefeld, 2023). Patients who achieve a sustained virologic response (SVR) after HCV therapy stop direct and indirect liver damage due to hepatitis C, reducing the risk of liver fibrosis progression and improving liver function, even in cirrhotic patients (Rockey and Friedman, 2021). Additionally, a substantial number of HCV-infected individuals develop extrahepatic complications of varying severity, which are effectively mitigated by HCV elimination with antiviral therapy (Ferreira et al., 2024; Salama et al., 2022).

However, despite HCV eradication, liver disease progression continues in some of them, increasing the risk of developing clinical events, such as HCC and death (Lynch and Russo, 2023), possibly due to molecular changes caused by chronic hepatitis C and associated with a greater risk of severe disease, as previously described in the liver tissue (Hamdane et al., 2019). In this regard, there is not much information about these pathophysiological mechanisms among patients with hepatitis C who achieve SVR (Rockey and Friedman, 2021; Kisseleva and Brenner, 2021).

Transcriptomics studies may be crucial to gain insight into the functioning of liver regeneration in these patients, and gene expression profiles could allow us to identify patients who will improve liver stiffness after SVR, constituting a clinically helpful non-invasive method. In this regard, liquid biopsy, such as peripheral blood, can be used to identify predictive biomarkers of liver disease due to the close connection between the liver and the immune system, also providing valuable information about the systemic immune response triggered by infection or liver injury.

### 1.1 Objective

We aimed to examine the association of peripheral blood mononuclear cells (PBMCs) gene expression in HIV/HCV-coinfected patients before HCV therapy with a pronounced decrease in the liver stiffness measurement (LSM) value after achieving SVR.

## 2 Methods

### 2.1 Patients

We conducted a retrospective study on 48 HIV/HCV-coinfected patients from the GESIDA 3603b (from July 2012 to March 2014, n = 16) and the ESCORIAL cohorts (from January to September 2015, n = 32) (see Appendix), which have been previously described (Medrano et al., 2021; Garcia-Broncano et al., 2020). All patients had advanced fibrosis or cirrhosis (LSM ≥9.5) and were on stable ART with undetectable plasma HIV viral load (<50 copies/ml). After sampling at baseline, all patients started anti-HCV treatment with interferon (IFN)/ribavirin or IFN/ribavirin/direct-acting antivirals (DAAs) in GESIDA 3603b and IFN-free DAAs therapy in ESCORIAL cohort patients. All patients achieved an SVR (undetectable HCV-RNA load 12/24 weeks after stopping anti-HCV treatment).

The inclusion criteria were: i) availability of a baseline PBMC sample at HIV HGM BioBank for RNA-seq; ii) available LSM data at baseline and 48 weeks after finishing the successful HCV treatment. The exclusion criteria were acute hepatitis C, hepatitis B virus coinfection, or HCC.

All patients signed the informed consent. Research Ethics Committee of the Instituto de Salud Carlos III (CEI PI 23_2011 and CEI PI 41_2014) approved this study, which was conducted according to the Declaration of Helsinki.

### 2.2 Clinical data

Epidemiological and clinical data were prospectively collected with an online form within each center, following data confidentiality requirements. LSM was assessed by transient elastography (FibroScan^®^, Echosens, Paris, France). Advanced fibrosis was defined by an LSM value between 9.5 and 12.4 kPa. Liver cirrhosis was defined by an LSM ≥12.5 kPa. Liver decompensation was defined by a history of clinically detectable ascites, variceal bleeding, or portosystemic encephalopathy.

### 2.3 Outcome variable

The main outcome variable was an LSM reduction greater than 50% (LSMred>50%) approximately 48 weeks after completing HCV treatment. To achieve this goal, patients were stratified into two groups (LSMred>50% vs. LSMred≤50%).

### 2.4 Samples, RNA sequencing, and bioinformatic pipeline

Peripheral venous blood samples were obtained by venipuncture in ethylenediaminetetraacetic acid tubes. On the same day of the extraction, clinical samples were sent to the HIV HGM BioBank, and PBMCs were processed by Ficoll-Paque density gradient and stored in liquid nitrogen (−180°C) until their use (Garcia-Merino et al., 2009). Samples were transferred to the National Center for Microbiology for subsequent analysis.

RNeasy Microkit (Qiagen, Hilden, Germany) was used to extract total RNA from PBMCs, according to the manufacturer’s instructions. NanoDrop 2000 Spectrophotometer (ThermoFisher) was used to quantify RNA concentration. RNA quality was evaluated by RNA Integrity Number (RIN) using 2100 Bioanalyzer RNA Nano assay (Agilent). Only samples with RIN>7.5 were selected for sequencing. Illumina’s TruSeq Stranded mRNA Sample Prep Kit v2 was used to synthesize libraries from 500 nanograms of total RNA per sample, which capture coding and noncoding polyadenylated RNAs. Specific barcodes were used for each sample for multiplex sequencing. The Illumina HiSeq2500, single read, 50 nt (1 × 50), was used for sequencing. Library synthesis and sequencing were performed at the Centre for Genomic Regulation in Barcelona (Spain). This process has been previously described in more detail (Brochado et al., 2021).

FastQC (v.0.11.8) was used for quality control, and Trimmomatic (v. 0.38) was used for adapter trimming to process raw sequencing data. GRCh38 was used as a reference genome to sequence alignment with STAR (v. 2.6.1day) and Subread’s featureCounts software (v. 1.6.4) for read count extraction at the gene level (Brochado et al., 2021).

### 2.5 Statistical analysis

Statistical analysis was performed using software R version 4.1.1 (R Foundation for Statistical Computing, Vienna, Austria). The Mann-Whitney U and Chi-Squared tests were used for descriptive analysis of continuous and discrete epidemiological and clinical variables, respectively. We analyzed the PBMCs gene expression differences (counts per million) between groups (LSMred>50% vs. LSMred≤50%) as follows. Firstly, RNA-Seq data was filtered and normalized with *DESeq2* package (v. 1.34.0), including only genes represented in at least 25% of the samples and with a minimum of 10 counts per gene. Next, we performed a generalized linear model (GLM) with a negative binomial distribution (*DESeq2* package in R).

Subsequently, a GLM with a negative binomial distribution and a stepwise covariate selection method (age, gender, HCV treatment, hepatic decompensation, and LSM value at baseline) was used to select the most relevant covariates and include them in the final model. Each step included these covariates based on the model’s (method forward) lowest AKAike information criteria (AIC).

Fold-change (FC) and level of significance (p-values) from the GLM test were adjusted for multiple testing using the false discovery rate (FDR) and Benjamini and Hochberg method (*q*-values). Significant differential expression (SDE) was defined as a gene with FC ≥ 2 in both directions and a q-value <0.2.

To identify robust gene signatures for discriminating between high and low levels of LSM reduction, we employed sparse Partial Least Squares-Discriminant Analysis (sPLS-DA), a supervised multivariate statistical technique. This analysis was performed on the previously identified SDEs transcripts from the adjusted GLM, using the R package mixOmics v6.3.2. The performance of the gene signature was assessed using the Area Under the Receiver Operating Characteristic Curve (AUROC). AUROC values were categorized as follows: outstanding (0.90–1.00), excellent (0.80–0.90), and acceptable (0.70–0.80). The Variable Importance in Projection (VIP) score was also calculated for each SDE (average of the three principal components) to identify the most influential features. A VIP score ≥1 indicated a significant contribution to the model. To further evaluate the discriminatory power of individual SDE transcripts, we conducted a univariate logistic regression analysis and subsequent calculation of the AUROC.

A GLM with a gamma distribution (log link) was employed to investigate the association between gene expression (independent variable) and baseline hepatic clinical parameters (dependent variable), adjusting for age, gender, HCV treatment, hepatic decompensation, and LSM.

### 2.6 Functional analysis

Database for Annotation, Visualization, and Integrated Discovery (DAVID) was used to identify enriched biological pathways.

### 2.7 Cell type analysis

The xCell web tool was used to infer type cell proportions from expression profiles (White et al., 2024; Aran et al., 2017). A GLM with a binomial distribution was employed to evaluate the association between xCell scores for PBMC cell types present in at least 60% of samples and LSMred>50%, adjusting for age, gender, HCV treatment, hepatic decompensation, and baseline LSM. Odds ratios (OR) and p-values were calculated to assess the strength and significance of these associations.

## 3 Results

### 3.1 Patient characteristics

The baseline clinical and epidemiological characteristics of the 48 HIV/HCV-coinfected patients included in the study are shown in Table 1. Seven patients (14.5%) achieved a reduction in LSM of more than 50%, and 41 (85.5%) had a reduction of less than 50%. At baseline, 72.9% were male, with a median age of 51 and a BMI of 23.8 kg/m^2^. A total of 51.2% and 81.2% had high alcohol intake and injection drug use, respectively. Co-morbidities related to extrahepatic manifestations were only observed in patients with an LSMred≤50%. HCV genotype 1 constituted the most frequently observed genotype (61.8%), followed by genotypes 3 (23.6%), 4 (10.9%), and 2 (1.8%). Regarding HCV treatment, 47.9% had received previous HCV therapy before being included in the study. During the study follow-up, 33.3% were treated with IFN-based therapy, while 66.7% were treated with IFN-free DAAs therapy. Besides, 14.6% had an LSM of 9.5–12.4 kPa, 20.8% of 12.5 kPa–19.9 kPa, and 64.6% of ≥20 kPa. Baseline characteristics were similar for LSMred>50% and LSMred≤50% groups, except for age (p = 0.002).

**TABLE 1:** Baseline characteristics of HIV/HCV-coinfected patients according to the change of LSM values.

	All	LSMred≤50%	LSMred>50%	*p*
No.	48	41	7	
Gender (male)	35 (72.9%)	31 (75.5%)	4 (57.1%)	0.578
Age (years)	51.1 [48.5–53.0]	51.8 [48.8–53.1]	47.0 [45.2–47.9]	0.002
BMI (kg/m^2^) (n = 47)	23.8 [21.3–25.5]	23.9 [21.4–25.6]	22.5 [21.6, 24.4]	0.650
BMI ≥25 (kg/m^2^) (n = 47)	15 (31.2%)	13 (31.7%)	2 (28.6%)	0.897
Alcohol consumed ever	26 (54.2%)	21 (51.2%)	5 (71.4%)	0.561
Intravenous drug user	39 (81.2%)	33 (80.5%)	6 (85.7%)	0.999
Co-morbidities (n = 39)				
Arterial hypertension	5 (12.8%)	5 (14.7%)	—	—
Diabetes Mellitus	6 (15.4%)	6 (17.6%)	—	—
Hyperlipidemia	2 (5.1%)	2 (5.9%)	—	—
Chronic renal insufficiency	1 (2.6%)	1 (2.9%)	—	—
HIV antiretroviral therapy				
NRTI + II-based	22 (45.8%)	19 (46.3%)	3 (42.9%)	0.999
NRTI + NNRTI-based	8 (16.7%)	7 (17.1%)	1 (14.3%)	0.999
NRTI + PI-based	4 (8.3%)	4 (9.8%)	—	—
PI-based	3 (6.1%)	3 (7.3%)	—	—
PI + II-based	4 (8.3%)	4 (9.8%)	—	—
Others	7 (14.6%)	4 (9.8%)	3 (42.9%)	0.087
HIV markers				
Nadir CD4+ T-cells	99 [70–181]	99 [65–176]	149 [115–254]	0.088
CD4+ T-cells/mm^3^	440 [243–763]	446 [246–749]	390 [261–900]	0.953
HCV therapy				
Previous HCV therapy	23 (47.9%)	20 (48.8%)	3 (42.9)	0.999
Baseline HCV Therapy				
IFN-based therapy	16 (33.3%)	13 (31.7%)	3 (42.9%)	0.858
IFN-free DAAs therapy	32 (66.7%)	28 (68.3%)	4 (57.1%)	0.858
HCV markers				
HCV genotype (n = 47)				
1	30 (61.8%)	25 (61.0%)	5 (71.4%)	0.978
2	1 (1.8%)	—	1 (14.3%)	—
3	11 (23.6%)	10 (24.4%)	1 (14.3%)	0.894
4	5 (10.9%)	5 (12.2%)	—	—
Log_10_ HCV-RNA (IU/mL)	6.32 [5.84, 6.59]	6.31 [5.97–6.72]	6.38 [5.52–6.45]	0.405
HCV-RNA > 850.000 IU/mL	35 (72.9%)	31 (75.6%)	4 (57.1%)	0.578
Liver disease				
Hepatic decompensation	18 (37.5%)	17 (41.5%)	1 (14.3%)	0.342
LSM at baseline (kPa)	25.7 [16.0–36.3]	22.3 [16.1–30.4]	48.0 [26.2–50.5]	0.085
9.5 to 12.4 kPa	7 (14.6%)	6 (14.6%)	1 (14.3%)	0.999
12.5 kPa to 19.9 kPa	10 (20.8%)	9 (22.0%)	1 (14.3%)	0.999
≥ 20 kPa	31 (64.6%)	26 (63.4%)	5 (71.4%)	0.999

**Statistics**: The values are expressed as the absolute number (percentage) and median (interquartile range). *P-values* were calculated by the Chi-squared test and the Mann-Whitney test.

**Abbreviations**: BMI, body mass index; HCV, hepatitis C virus; HIV, human immunodeficiency virus; NRTI, nucleoside analogue HIV reverse transcriptase inhibitor; II, HIV integrase inhibitor; NNRTI, non-nucleoside analogue HIV reverse transcriptase inhibitor; PI, HIV protease inhibitor; LSM, liver stiffness measurement; HCV-RNA, viral load of hepatitis C.

Hematological and biochemical data at both time points are detailed in Supplementary Table S1. A statistically significant decrease in bilirubin and transaminase levels was observed, concurrent with a significant increase in neutrophil count, albumin, total cholesterol, and LDL.

### 3.2 Differential expression analysis

We obtained an average of 23.5 million reads per sample, and 99% of reads mapped to the reference genome. A total of 60,623 different genes were identified, but only 14,858 fulfilled the filtering criteria for subsequent analysis (see methods).

**FIGURE 1:**
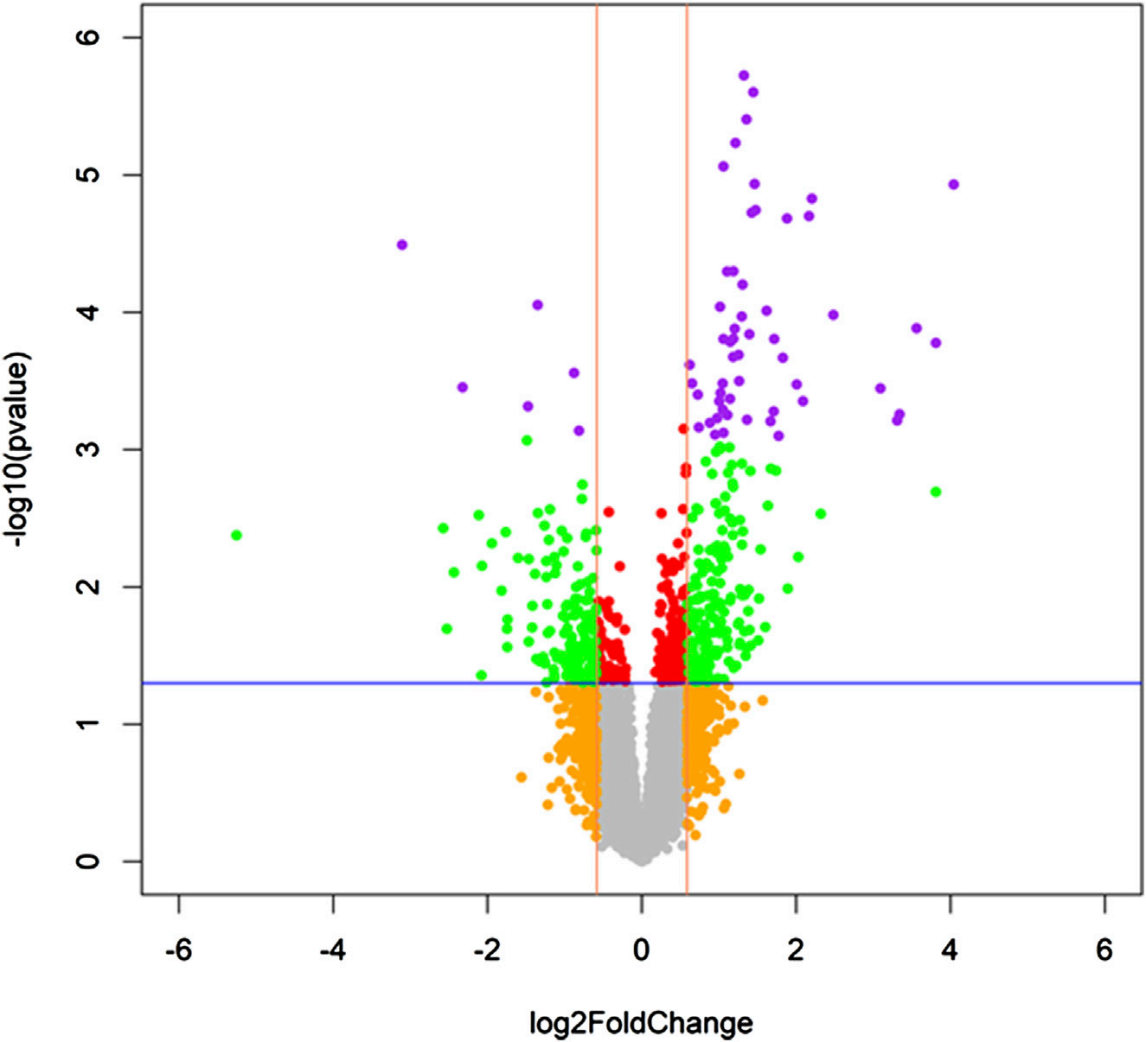
Volcano plot of significant differentially expressed genes in PBMCs between patients who reduced LSM values > 50% (LSMred>50%) and those who did not (LSMred≤50%) after 48 weeks of finishing the HCV treatment. Statistics: The volcano plot is depicted with each transcript’s fold change (FC) calculated by an unadjusted GLM test. The significance level was corrected for multiple tests using the false discovery rate (FDR) with the Benjamini and Hochberg method. Significant upregulated and downregulated transcripts are shown as purple circles (q-value <0.2). Abbreviations: LSM, liver stiffness measurement; FC, fold-change; FDR, false discovery rate; PBMCs, peripheral blood mononuclear cells.

A total of 52 SDE transcripts between the LSMred>50% and LSMred≤50% groups were identified (Figure 1). Subsequent adjusted GLM analysis revealed 47 SDE transcripts, with 42 upregulated and 5 downregulated in the LSMred>50% group (Table 2). Notably, fifteen of these upregulated genes were significantly enriched in the Kyoto Encyclopedia of Genes and Genomes (KEGG) pathways of cell cycle and progesterone-mediated oocyte maturation, respectively (q-value <0.05).

**TABLE 2:** Summary of significant differentially expressed genes (absolute fold-change ≥2; q-value ≤0.05) in peripheral blood mononuclear cells between HIV/HCV-coinfected patients who reduced LSM values >50% (LSMred>50%) and those who did not (LSMred≤50%) after 48 weeks after completing HCV treatment.

Gene symbol	FC	log_2_ FC	p-value *	q-value **
HBB	50.62	5.66	3.81E-10	9.92E-09
HBA1	46.50	5.54	3.07E-11	1.60E-09
SLC4A1	29.01	4.86	4.49E-08	5.83E-07
HBA2	28.40	4.83	2.01E-08	3.49E-07
HBD	24.53	4.62	2.65E-07	1.97E-06
CA1	18.19	4.19	2.05E-07	1.78E-06
IGLV2-18	5.59	2.48	1.33E-04	2.23E-04
CDC20	4.50	2.17	1.86E-05	4.21E-05
AC092490.1	4.01	2.00	3.40E-04	4.14E-04
DLGAP5	3.70	1.89	1.68E-05	3.96E-05
IGHG2	3.63	1.86	1.11E-03	1.28E-03
IGLV2-11	3.63	1.86	4.24E-04	5.01E-04
IGLV2-23	3.55	1.83	2.98E-04	3.78E-04
IGLV2-14	3.52	1.82	2.78E-04	3.61E-04
HJURP	3.39	1.76	1.20E-07	1.25E-06
UBE2C	3.33	1.74	8.61E-06	2.71E-05
SNCA	3.29	1.72	2.08E-04	3.00E-04
IGLV4-69	2.95	1.56	3.78E-03	4.02E-03
PKMYT1	2.78	1.47	1.20E-05	3.13E-05
OSBP2	2.68	1.42	1.17E-05	3.13E-05
CEACAM1	2.61	1.38	6.00E-03	6.24E-03
MKI67	2.47	1.30	6.67E-05	1.27E-04
RRM2	2.46	1.30	1.70E-04	2.60E-04
TPX2	2.46	1.30	2.40E-06	9.58E-06
GTSE1	2.44	1.29	1.29E-05	3.19E-05
KIF2C	2.41	1.27	7.24E-06	2.69E-05
CCNA2	2.39	1.25	1.58E-04	2.56E-04
CDC6	2.36	1.24	4.00E-05	8.31E-05
CDCA5	2.36	1.24	1.20E-05	3.13E-05
E2F8	2.35	1.23	2.74E-04	3.61E-04
CDKN3	2.32	1.21	3.75E-05	8.13E-05
BUB1	2.28	1.19	1.28E-04	2.21E-04
MCM10	2.24	1.16	1.44E-06	7.47E-06
CDT1	2.23	1.15	8.86E-06	2.71E-05
MELK	2.22	1.15	7.06E-05	1.27E-04
CDC25A	2.21	1.14	1.95E-04	2.90E-04
FOXM1	2.19	1.13	2.77E-04	3.61E-04
CDC45	2.19	1.13	2.32E-04	3.26E-04
CCNB1	2.14	1.10	8.05E-06	2.71E-05
CKAP2L	2.08	1.06	7.09E-05	1.27E-04
NCAPG	2.08	1.06	2.15E-06	9.31E-06
TOP2A	2.02	1.01	6.91E-05	1.27E-04
FAT4	0.46	−1.11	1.24E-02	1.26E-02
NHLRC1	0.30	−1.72	3.69E-07	2.40E-06
DPY19L1P1	0.19	−2.36	3.42E-04	4.14E-04
MYOM2	0.07	−3.79	2.13E-06	9.31E-06
MTRNR2L1	0.00	−7.89	7.45E-07	4.30E-06

**Statistics**: Values are expressed as fold-change (FC) and its log2. (*), raw p-values; (**), p-values corrected using the Benjamini and Hochberg procedure. Data were calculated using a GLM with a negative binomial distribution and a stepwise covariate selection method (see the statistical analysis section).

**Abbreviations**: HCV, hepatitis C virus; HIV, human immunodeficiency virus; FC, fold change.

Finally, the analysis of cell type composition, based on expression profiles (Supplementary Figure S1 **)**, showed variability across samples. However, no significant differences were observed between cell type proportion and a LSMred>50% (Supplementary Table S2).

### 3.3 Discriminant analysis

**FIGURE 2:**
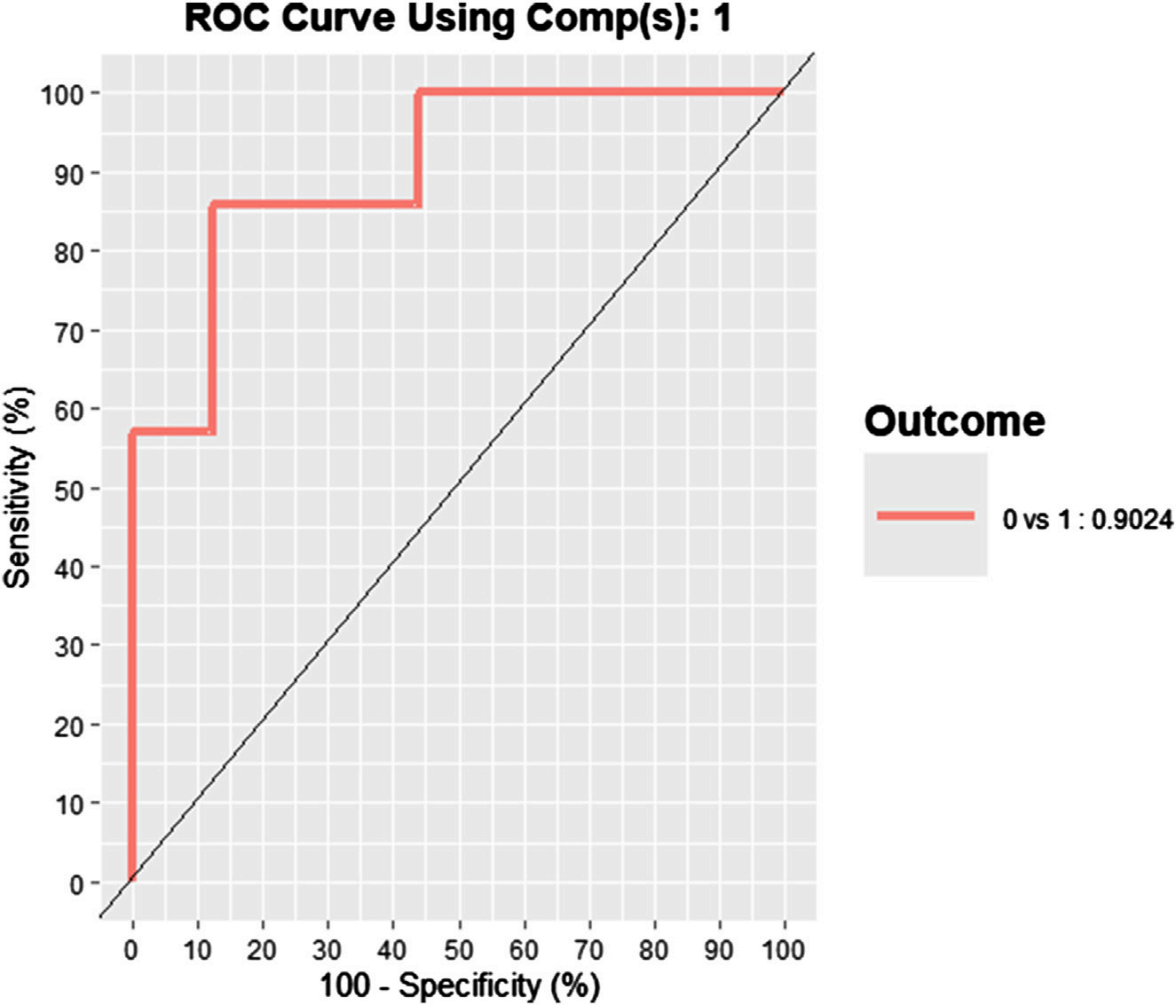
Multivariate analysis of 47 SDE transcripts from PBMCs to discriminate between patients with a significant reduction in liver stiffness measurement (LSM >50%) and those with a minimal or no reduction (LSM ≤50%) 48 weeks post-HCV treatment. Statistics: Data analysis was performed using a Sparse Partial Least Squares Discriminant Analysis (sPLS-DA), the ROC curve was plotted, and AUROC was calculated. Abbreviations: ROC, Receiver operating characteristic; AUROC, area under the ROC.

The sPLS-DA performed on the 47 SDE genes at baseline yielded an AUROC of 0.902, demonstrating excellent discrimination between the LSMred>50% and LSMred≤50% groups (Figure 2). Notably, seven genes—*NCAPG*, *NHLRC1*, *AC092490.1*, *MCM10*, *OSBP2*, *CDKN3*, and *FAT4*—exhibited VIP scores ≥1.5, indicating their significant contribution to the model (Supplementary Table S3).

Logistic regression analyses were performed to evaluate the individual discriminatory power of the seven SDE genes with VIP scores ≥1.5. Two genes, *NCAPG* and *NHLRC1*, demonstrated excellent individual discrimination ability, with AUROC values of 0.908 (95%CI = 0.815–0.999) and 0.879 (95%CI = 0.753–0.999), respectively. A logistic regression model incorporating the expression values of *NCAPG* and *NHLRC1* jointly further improved the discriminatory performance, achieving an AUROC of 0.955.

When evaluating the association between *NCAPG* and *NHLRC1* gene expression and laboratory parameters at baseline, we observed that albumin had a negative and positive association with *NCAPG* and *NHLRC1* gene expression, respectively (Supplementary Table S4). In contrast, no significant association was identified for LSM, platelets, and transaminases.

## 4 Discussion

In this preliminary study, we analyzed the PBMCs transcriptome profile of HIV/HCV-coinfected patients to identify those genes associated with a liver fibrosis regression (LSMred>50%) after achieving HCV clearing with HCV therapy. We found 47 SDE genes, among which *NCAPG* and *NHLRC1* stood out for their excellent predictive capacity. Thus, we described for the first time the transcriptomic profiling at baseline associated with a pronounced LSM reduction and, thus, probably associated with a lower risk of future liver-related complications. However, given the small size of our sample, confirming our findings in a validation cohort is necessary to determine the relevance of this research.

Achieving SVR reduces complications of liver disease. In this regard, previous data have described a reduction of liver disease, measured by LSM, after HCV eradication (Knop et al., 2021), but a significant risk of cirrhosis progression remains in some patients (Rockey and Friedman, 2021). Here, we observed that only 14.5% of the patients had a pronounced reduction of LSM (at least 50%) after HCV eradication. Fibrosis regression appears to peak approximately 1 year after achieving SVR (Chekuri et al., 2016), but the exact causes are unknown. Therefore, it is vital to investigate biomarkers that identify patients who achieve adequate regression of liver fibrosis after eliminating HCV with antiviral treatment.

This study found a genetic signature of 47 SDE genes at baseline associated with liver fibrosis regression (LSMred>50%), which showed implications in the biological pathways “cell cycle” and “progesterone-mediated oocyte maturation”. HCV infection causes alterations in different biological processes, such as immune activation, senescence, and chronic inflammation (Naggie, 2017), which could impact cell cycle regulation. The altered gene expression in this pathway could be related to a better gene expression profile of patients who reduced the LSM value (Hora and Wuestefeld, 2023). Previous studies have observed an association between the progesterone-mediated oocyte maturation pathway and the development and progression of HCC (Chen et al., 2018; Yang et al., 2023; Liu et al., 2021), suggesting a potential link between reproductive hormones and liver carcinogenesis. This association may be partially explained by progesterone’s influence on hepatic lipid metabolism. Specifically, progesterone has been shown to increase hepatic lipid content and lipid levels through PR-B-mediated lipogenesis (Jeong et al., 2024), a process that can contribute to non-alcoholic fatty liver disease (NAFLD) and its progression to HCC. However, the precise mechanisms underlying progesterone’s role in HCC remain unclear, and further studies are needed to fully elucidate its function and potential therapeutic implications.

It is noteworthy that several hemoglobin subunit genes, such as alpha (HBA), beta (HBB), and delta (HBD), were identified. Hemoglobin plays crucial roles in establishing host resistance against pathogens and regulating innate immune responses and its expression is not unique to erythrocytes. The beta subunit is a pleiotropic regulator of RIG-I/MDA5-mediated antiviral responses, highlighting the importance of the intercellular microenvironment, including the redox state, in regulating antiviral innate immune responses (Yang et al., 2019). Previous studies have reported that changes in gene expression of these genes are associated with stress caused by several non-infectious diseases (Chen et al., 2022; Derakhshani et al., 2021) and infectious ones such as COVID-19 (Eltobgy et al., 2024) or sepsis (Leite et al., 2019). Also, hemoglobin can reduce oxidative stress in certain pathologies, such as non-alcoholic steatohepatitis (NASH) (Liu et al., 2011). Therefore, the increased expression of hemoglobin subunit genes in LSMred>50% patients could be related to an attempt to buffer the excess oxidative stress of these patients.

Among these 47 SDE-identified genes, *NCAPG* and *NHLRC1* were those that best at discriminating patients who achieved regression of liver fibrosis (LSMred>50%). The *NCAPG* encodes a subunit of condensin I complex involved in chromosome condensation, participating in the mitotic cell division. The NCAPG protein plays a special role in the pathogenesis of several tumors since it promotes abnormal functions in processes like cell proliferation, apoptosis, or cell cycle (Cai et al., 2022). However, NCAPG also seems essential for stem cell maintenance (Lai et al., 2018), so its higher expression in patients who reach LSMred>50% may be necessary for liver regeneration. The *NHLRC1* encodes the malin protein, an E3-ubiquitin ligase incorporating lysine 63 (K63)-linked polyubiquitin chains, promoting autophagic and degradation of ubiquitylated targets (Garcia-Gimeno et al., 2018). Although the molecular mechanism is not known in detail, K63-linked ubiquitination regulates multiple signaling pathways, including the regulation of stem cell biology (Werner et al., 2017). Therefore, it cannot be ruled out that the low *NHLRC1* expression indicates greater liver regenerative capacity. Thus, the expression levels of those two genes could be clinically used as putative biomarkers of pronounced regression of liver fibrosis after achieving SVR with HCV therapy.

### 4.1 Study limitations

This study has some limitations to take into account. Firstly, the limited sample size may affect the validity of the study and the statistical power of the analysis. Secondly, the observational design may have introduced biases, which we have tried to correct by adjusting the GLM analysis for the most relevant covariables. Third, no additional sample was available to validate the expression of the genes of interest by qRT-PCR. However, we evaluated gene expression using RNA-seq, an accepted and quite accurate quantitative method (Consortium, 2014). Fourth, liver biopsy fibrosis data were not available, but we have used transient elastography, a validated non-invasive method to evaluate liver fibrosis in HIV/HCV-coinfected patients (Resino et al., 2012). Fifth, further validation in independent cohorts is necessary to confirm the predictive value of *NCAPG* and *NHLRC1*.

### 4.2 Conclusions

A pre-treatment gene expression signature of 47 SDE genes in PBMCs was associated with liver fibrosis regression (LSMred>50%) after achieving HCV clearing with HCV therapy in HIV/HCV-coinfected patients, where two SDE genes (*NCAPG* and *NHLRC1*) showed the greatest predictive capacity, which could be used as a non-invasive marker of liver fibrosis regression in patients co-infected with HIV/HCV.

## Data Availability

The datasets presented in this study can be found in online repositories. The names of the repository/repositories and accession number(s) can be found below: https://www.ebi.ac.uk/, accession number E-MTAB-12251.

## Abbreviations

AIC, AKAike information criteria; AUROC, Area under receiver operating characteristic curve; cART, Combination antiretroviral therapy; DAVID, Database for Annotation, Visualization and Integrated Discovery; DAAs, Direct-acting antivirals; FDR, False discovery rate; FC, Fold-change; GLM, Generalized linear models; HVPG, Hepatic venous pressure gradient; HCV, Hepatitis C virus; HCC, Hepatocellular carcinoma; HGM, Hospital Gregorio Marañon; HIV, Human immunodeficiency virus; IFN, Interferon; KEGG, Kyoto encyclopedia of genes and genomes; LSM, Liver stiffness measurement; NHLRC1, NHL repeat containing E3 ubiquitin protein ligase 1; NCAPG, Non-SMC Condensin I Complex Subunit G; OR, Odds ratio; PBMCs, Peripheral blood mononuclear cells; RIN, RNA integrity number; SDE, Significantly differentially expressed; SVR, Sustained virological response.
