# Supplementary material for: PBMCs gene expression predicts liver fibrosis regression after successful HCV therapy in HIV/HCV-coinfected patients
Source: Front Pharmacol. 2025 Jan 22;15:1436198. doi: 10.3389/fphar.2024.1436198 (PMC11794839; doi:10.3389/fphar.2024.1436198)
Supplement: Supplementary file 1 [file Table1.docx]

**Supplementary Table 1**. Hematological and biochemical characteristics of patients at baseline and follow-up.

|  | **Baseline** | **Follow-up** | ***p-value*** |
| --- | --- | --- | --- |
| **No.** | 48 | 48 |  |
| **Neutrophils (10^6mL)** | 2.57 [1.70, 3.26] | 3.19 [2.18, 4.10] | **0.036** |
| **Platelets count (10^9/L)** | 91.50 [53.00, 119.50] | 101.00 [69.50, 144.00] | 0.212 |
| **Albumin (g/dL)** | 4.00 [3.70, 4.30] | 4.40 [4.10, 4.60] | **0.001** |
| **Creatinine (mg/dL)** | 0.80 [0.70, 0.92] | 0.84 [0.77, 1.00] | 0.230 |
| **Bilirubin (mg/dL)** | 0.96 [0.67, 1.38] | 0.70 [0.50, 1.04] | **0.010** |
| **Glycemia (mg/dL)** | 96.50 [87.00, 105.00] | 93.00 [85.50, 101.50] | 0.370 |
| **TG (mmol/L)** | 115.00 [79.50, 153.00] | 123.00 [82.00, 185.50] | 0.443 |
| **Total cholesterol** | 143.00 [124.25, 180.75] | 163.00 [143.00, 188.50] | **0.018** |
| **HDL (mmol/L)** | 41.00 [32.00, 55.00] | 40.00 [35.00, 58.00] | 0.507 |
| **LDL (mmol/L)** | 70.50 [56.00, 103.25] | 89.00 [78.00, 108.00] | **0.019** |
| **AST (UI/L)** | 72.00 [42.50, 104.00] | 30.00 [23.50, 35.75] | **<0.001** |
| **ALT (UI/L)** | 63.00 [41.00, 92.00] | 22.00 [17.50, 31.50] | **<0.001** |

**Statistics:** The values are expressed as median (interquartile range). *P-values* were calculated by the Wilcoxon test. **Abbreviations** AST, aspartate aminotransferase; ALT, alanine aminotransferase; HDL, high-density lipoprotein; LDL, low-density lipoprotein; TG, triglycerides.
