# Supplementary material for: PBMCs gene expression predicts liver fibrosis regression after successful HCV therapy in HIV/HCV-coinfected patients
Source: Front Pharmacol. 2025 Jan 22;15:1436198. doi: 10.3389/fphar.2024.1436198 (PMC11794839; doi:10.3389/fphar.2024.1436198)
Supplement: Supplementary file 2 [file Table2.docx]

**Supplementary Table 2**. Association between xCell scores for each of the major PBMC cell types estimated by the xCell algorithm and LSM reduction values >50%(LSMred>50%).

| **Cell types** | **aOR (95%CI)** | ***p*-value** |
| --- | --- | --- |
| **B-cells** | 1.21 (0.68-2.14) | 0.514 |
| **CD4+ T-cells** | 1.23 (0.40-3.79) | 0.723 |
| **CD4+ memory T-cells** | 1.20 (0.146-3.08) | 0.710 |
| **CD4+ naive T-cells** | 1.42 (0.25-8.15) | 0.696 |
| **CD8+ T-cells** | 1.13 (0.62-2.07) | 0.691 |
| **CD8+ naive T-cells** | 1.02 (0.20-5.20) | 0.977 |
| **Naive B-cells** | 1.17 (0.72-1.93) | 0.524 |
| **Memory B-cells** | 1.22 (0.67-2.22) | 0.511 |
| **Dendritic cells** | 1.20 (0.71-2.04) | 0.501 |
| **Monocytes** | 1.20 (0.92-1.55) | 0.173 |
| **NK cells** | 0.96 (0.68-1.35) | 0.826 |

**Statistics:** Associations were calculated using a generalized linear model (GLM) with a binomial distribution. Significant differences are shown in bold (p-value<0.05).

**Abbreviations:** aOR, adjusted odds ratio; 95%CI, 95% of confidence interval; CD, cluster of differentiation; p, level of significance; q, corrected level of significance; LSM, liver stiffness measure; NK, natural killer.
