# Supplementary material for: PBMCs gene expression predicts liver fibrosis regression after successful HCV therapy in HIV/HCV-coinfected patients
Source: Front Pharmacol. 2025 Jan 22;15:1436198. doi: 10.3389/fphar.2024.1436198 (PMC11794839; doi:10.3389/fphar.2024.1436198)
Supplement: Supplementary file 3 [file Table3.docx]

**Supplementary Table 3**. Summary of variable importance in projection (VIP) of significant differentially expressed (SDE) genes in peripheral blood mononuclear cells between HIV/HCV-coinfected patients who reduced LSM values >50% (LSMred>50%) and those who did not (LSMred≤50%) after 48 weeks after completing HCV treatment.

| **Gene symbol** | **VIP** |
| --- | --- |
| **NCAPG** | 1.93 |
| **NHLRC1** | 1.85 |
| **AC092490.1** | 1.78 |
| **MCM10** | 1.67 |
| **OSBP2** | 1.67 |
| **CDKN3** | 1.66 |
| **FAT4** | 1.59 |
| **DPY19L1P1** | 1.39 |
| **CEACAM1** | 1.35 |
| **HBA1** | 1.32 |
| **HBA2** | 1.28 |
| **CCNB1** | 1.28 |
| **SLC4A1** | 1.17 |
| **CA1** | 1.12 |
| **MELK** | 1.07 |
| **KIF2C** | 1.07 |
| **HBB** | 1.06 |
| **TPX2** | 1.04 |
| **HBD** | 0.87 |
| **IGLV4-69** | 0.84 |
| **CKAP2L** | 0.83 |
| **HJURP** | 0.80 |
| **CDT1** | 0.80 |
| **IGLV2-18** | 0.78 |
| **SNCA** | 0.78 |
| **RRM2** | 0.73 |
| **IGLV2-23** | 0.70 |
| **CDCA5** | 0.63 |
| **PKMYT1** | 0.59 |
| **GTSE1** | 0.59 |
| **CDC45** | 0.58 |
| **DLGAP5** | 0.53 |
| **IGLV2-14** | 0.50 |
| **MYOM2** | 0.49 |
| **TOP2A** | 0.49 |
| **CDC20** | 0.45 |
| **MTRNR2L1** | 0.42 |
| **MKI67** | 0.42 |
| **CDC25A** | 0.41 |
| **UBE2C** | 0.40 |
| **FOXM1** | 0.37 |
| **BUB1** | 0.37 |
| **E2F8** | 0.37 |
| **IGLV2-11** | 0.34 |
| **CCNA2** | 0.33 |
| **CDC6** | 0.32 |
| **IGHG2** | 0.26 |

**Statistics**: Data were calculated using a sPLS-DA (see the statistical analysis section).

**Abbreviations**: HCV, hepatitis C virus; HIV, human immunodeficiency virus; VIP, variable importance in projection (VIP), SDE significant differentially expressed.
