# Supplementary material for: PBMCs gene expression predicts liver fibrosis regression after successful HCV therapy in HIV/HCV-coinfected patients
Source: Front Pharmacol. 2025 Jan 22;15:1436198. doi: 10.3389/fphar.2024.1436198 (PMC11794839; doi:10.3389/fphar.2024.1436198)
Supplement: Supplementary file 4 [file Table4.docx]

**Supplementary Table 4.** Association between *NCAPG* and *NHLRC1* with clinical parameters of interest.

|  | ***NCAPG*** | | | ***NHLRC1*** | |
| --- | --- | --- | --- | --- | --- |
| **Laboratory parameters** | **aAMR (95%CI)** | ***p*-value** | **aAMR (95%CI)** | | ***p*-value** |
| **LSM** | 1.12 (0.84-1.50) | 0.432 | 0.91 (0.73-1.12) | | 0.384 |
| **Platelets** | 0.89 (0.66-1.21) | 0.466 | 1.14 (0.91-1.42) | | 0.271 |
| **Albumin** | 0.90 (0.92-0.97) | **0.013** | 1.11 (1.05-1.18) | | **<0.001** |
| **AST** | 0.88 (0.58-1.35) | 0.568 | 0.94 (0.69-1.28) | | 0.690 |
| **ALT** | 0.81 (0.47-1.38) | 0.438 | 0.95 (0.63-1.42) | | 0.805 |

**Statistics:** Associations were calculated using a Generalized Linear Model (GLM) with a gamma distribution. Significant differences are shown in bold (p-value<0.05).

**Abbreviations:** AMR, the ratio of the arithmetic means; 95%CI, 95% of confidence interval; p, level of significance; q, corrected level of significance; LSM, liver stiffness measure; AST, aspartate aminotransferase; ALT, alanine aminotransferase.
