# Supplementary material for: PBMCs gene expression predicts liver fibrosis regression after successful HCV therapy in HIV/HCV-coinfected patients
Source: Front Pharmacol. 2025 Jan 22;15:1436198. doi: 10.3389/fphar.2024.1436198 (PMC11794839; doi:10.3389/fphar.2024.1436198)
Supplement: Supplementary file 5 [file DataSheet1.docx]

# Appendix

**The GESIDA 3603b Cohort Study Group**

***Hospital General Universitario Gregorio Marañón, Madrid:*** A Carrero, P Miralles, JC López, F Parras, B Padilla, T Aldamiz-Echevarría, F Tejerina, C Díez, L Pérez-Latorre, C Fanciulli, I Gutiérrez, M Ramírez, S Carretero, JM Bellón, J Bermejo, and J Berenguer.

***Hospital Universitario La Paz, Madrid:*** V Hontañón, JR Arribas, ML Montes, I Bernardino, JF Pascual, F Zamora, JM Peña, F Arnalich, M Díaz, J González-García.

***Hospital de la Santa Creu i Sant Pau, Barcelona:*** P Domingo, JM Guardiola.

***Hospital Universitari Vall d'Hebron, Barcelona:*** E Van den Eynde, M Pérez, E Ribera, M Crespo.

***Hospital Universitario Ramón y Cajal, Madrid:*** JL Casado, F Dronda, A Moreno, MJ Pérez-Elías, MA Sanfrutos, S Moreno, C Quereda.

***Hospital Universitario Príncipe de Asturias, Alcalá de Henares:*** A Arranz, E Casas, J de Miguel, S Schroeder, J Sanz.

***Hospital Universitario de La Princesa, Madrid:*** J Sanz, I Santos.

***Hospital Donostia, San Sebastián:*** MJ Bustinduy, JA Iribarren, F Rodríguez-Arrondo, MA Von-Wichmann.

***Hospital Clínico San Carlos, Madrid:*** J Vergas, MJ Téllez.

**Hospital Universitario San Cecilio, Granada**: D. Vinuesa, L. Muñoz, and J. Hernández-Quero.

***Hospital Clínico Universitario, Valencia:*** A Ferrer, MJ Galindo.

***Hospital General Universitario, Valencia:*** L Ortiz, E Ortega.

***Hospital Universitari La Fe, Valencia:*** M Montero, M Blanes, S Cuellar, J Lacruz, M Salavert, J López-Aldeguer.

***Hospital Universitario de Getafe, Getafe:*** G Pérez, G Gaspar.

***Fundación SEIMC-GESIDA, Madrid:*** M Yllescas, P Crespo, E Aznar, H Esteban

**The ESCORIAL study group**

***Hospital General Universitario Gregorio Marañón*** (Madrid, Spain): Cristina Díez, Luis Ibáñez, Leire Pérez-Latorre, Diego Rincón, Teresa Aldámiz-Echevarría, Vega Catalina, Pilar Miralles, Teresa Aldámiz-Echevarría, Francisco Tejerina, María C Gómez-Rico, Esther Alonso, José M Bellón, Rafael Bañares, and Juan Berenguer.

***Hospital Universitario La Paz/IdiPAZ*** (Madrid, Spain): José Arribas, José I Bernardino, Ana Delgado, Carmen Busca, Javier García-Samaniego, Víctor Hontañón, Luz Martín-Carbonero, Rafael Micán, María L Montes-Ramírez, Victoria Moreno, Antonio Olveira, Ignacio Pérez-Valero, Eulalia valencia, and Juan González-García.

***Hospital Universitario Puerta de Hierro*** (Madrid, Spain): Elba Llop and José Luis Calleja.

***Hospital Universitario Ramón y Cajal*** (Madrid, Spain): Javier Martínez and Agustín Albillos.

***Fundación SEIMC/GeSIDA*** (Madrid, Spain): Marta de Miguel, María Yllescas, and Herminia Esteban.
