# Supplementary material for: PBMCs gene expression predicts liver fibrosis regression after successful HCV therapy in HIV/HCV-coinfected patients
Source: Front Pharmacol. 2025 Jan 22;15:1436198. doi: 10.3389/fphar.2024.1436198 (PMC11794839; doi:10.3389/fphar.2024.1436198)

Supplementary Figure 1. Visualization of xCell scores for each of the major PBMC cell types estimated by the xCell algorithm in baseline samples.

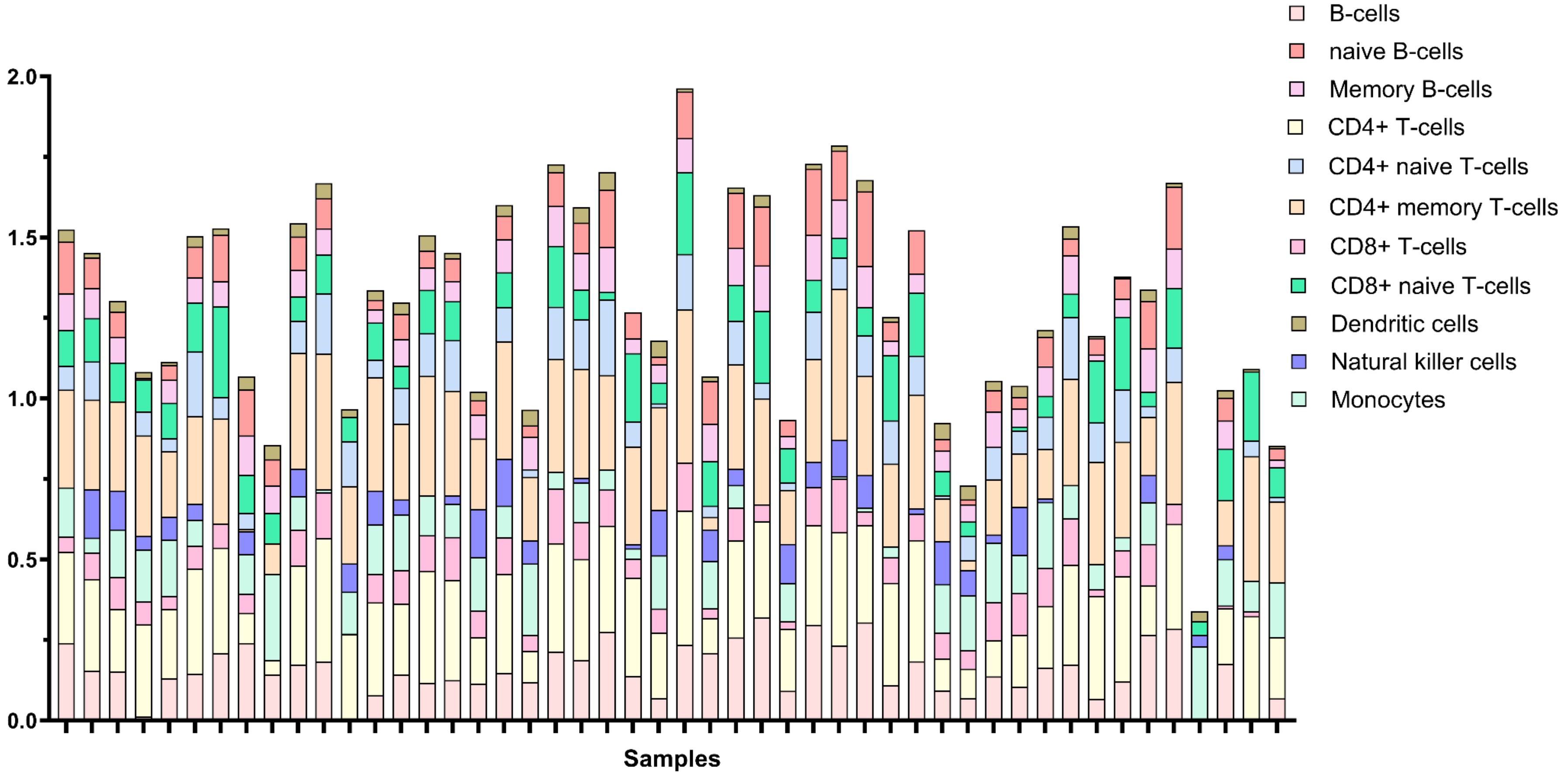

Supplement: Supplementary file 6 [file Image1.pdf]
